# The human arthritic hip joint is a source of mesenchymal stromal cells (MSCs) with extensive multipotent differentiation potential

**DOI:** 10.1186/s12891-020-03340-z

**Published:** 2020-05-13

**Authors:** Mike Wagenbrenner, Tizian Heinz, Konstantin Horas, Axel Jakuscheit, Joerg Arnholdt, Marietta Herrmann, Maximilian Rudert, Boris M. Holzapfel, Andre F. Steinert, Manuel Weißenberger

**Affiliations:** 1 Department of Orthopaedic Surgery, University of Wuerzburg, Koenig-Ludwig-Haus, Brettreichstr. 11, 97074 Wuerzburg, Germany; 2 Bernhard-Heine-Center for Locomotion Research, University of Wuerzburg, Wuerzburg, Germany; 3 IZKF Research Group Tissue Regeneration in Musculoskeletal Disease, University Clinics Wuerzburg, Wuerzburg, Germany; 4 Department of Orthopaedic, Trauma, Shoulder and Arthroplasty Surgery, Rhön-Klinikum Campus Bad Neustadt, Von-Guttenberg-Str. 11, 97616 Bad Neustadt, Germany

**Keywords:** Hip joint, Osteoarthritis, MSCs, Cartilage regeneration, Tissue engineering, Ligamentum capitis femoris, Joint capsule, Bone marrow

## Abstract

**Background:**

While multiple in vitro studies examined mesenchymal stromal cells (MSCs) derived from bone marrow or hyaline cartilage, there is little to no data about the presence of MSCs in the joint capsule or the ligamentum capitis femoris (LCF) of the hip joint. Therefore, this in vitro study examined the presence and differentiation potential of MSCs isolated from the bone marrow, arthritic hyaline cartilage, the LCF and full-thickness samples of the anterior joint capsule of the hip joint.

**Methods:**

MSCs were isolated and multiplied in adherent monolayer cell cultures. Osteogenesis and adipogenesis were induced in monolayer cell cultures for 21 days using a differentiation medium containing specific growth factors, while chondrogenesis in the presence of TGF-ß1 was performed using pellet-culture for 27 days. Control cultures were maintained for comparison over the same duration of time. The differentiation process was analyzed using histological and immunohistochemical stainings as well as semiquantitative RT-PCR for measuring the mean expression levels of tissue-specific genes.

**Results:**

This in vitro research showed that the isolated cells from all four donor tissues grew plastic-adherent and showed similar adipogenic and osteogenic differentiation capacity as proven by the histological detection of lipid droplets or deposits of extracellular calcium and collagen type I. After 27 days of chondrogenesis proteoglycans accumulated in the differentiated MSC-pellets from all donor tissues. Immunohistochemical staining revealed vast amounts of collagen type II in all differentiated MSC-pellets, except for those from the LCF. Interestingly, all differentiated MSCs still showed a clear increase in mean expression of adipogenic, osteogenic and chondrogenic marker genes. In addition, the examination of an exemplary selected donor sample revealed that cells from all four donor tissues were clearly positive for the surface markers CD44, CD73, CD90 and CD105 by flow cytometric analysis.

**Conclusions:**

This study proved the presence of MSC-like cells in all four examined donor tissues of the hip joint. No significant differences were observed during osteogenic or adipogenic differentiation depending on the source of MSCs used. Further research is necessary to fully determine the tripotent differentiation potential of cells isolated from the LCF and capsule tissue of the hip joint.

## Background

Tissue loss as well as organ failure due to traumatic, congenital or acquired diseases pose a substantial health threat in our modern society [1–3]. Despite medical innovations enabling the transplantation of allogenic tissue and organs, there is, according to the World Health Organization (WHO), a substantial lack of potential donors [1, 4]. More importantly, constant increases in life expectancy have led to a skyrocketing of degenerative diseases which generate both extremly high expenses for our health care systems and a growing amount of patients that could benefit from progress in the area of regenerative medicine [2, 3, 5]. These challenges have led to constant new research in the field of Tissue Engineering (TE) [6, 7]. TE aims to regenerate or maintain tissue function through the combined use of living cells with biomaterials and specific growth factors or signalling pathways to guide or maintain cell fate [7].

Disorders of the locomotive system, such as soft tissue defects, diseases of the skeletal systeme and loss of articular cartilage as seen in osteoarthritis, majorly contribute to the rise of chronic, degenerative diseases that can benefit from TE [8, 9]. Especially the poor capacity for self-regeneration in articular cartilage has been the subject of various in vitro and in vivo research [8, 10]. While current techniques for cell-based cartilage repair such as autologous chondrocyte transplantation (ACT), have shown beneficial results in the short-term, there is still no reliable technique to restore long-lasting hyaline cartilage in vivo [11–13]. In addition, the harvest of mature, autologous-derived cells from healthy tissue causes iatrogen damage at the extraction site and often requires invasive surgery while only offering a small yield of cells [12].

In contrast to mature cells mesenchymal stromal cells (MSCs) offer an extensive multipotent differentiation potential and vast amounts of MSCs can be isolated from easy accessible tissues minimizing donor site morbidity [14]. Furthermore, in contrast to stem cells they raise fewer ethical questions and carry less risk of cancer formation after transplantation [15, 16]. The minimal requirements to be met by cells in order to be viewed as MSCs were defined by the International Society for Cellular Therapy (ISCT) in 2006 [17, 18]. These criteria include the expression (≥ 95% positive) of the surface molecules CD73, CD90 and CD105 as well as the lack of expression (≤ 2% positive) of haematopoetic cell markers. In addition, MSCs must grow plastic-adherent and show the ability to differentiate into osteoblasts, adipocytes and chondroblasts in vitro which is why they are frequently used for engineering osteochondral or soft tissue [17, 18].

When comparing sources of MSCs, bone marrow-derived MSCs (BM-MSCs) are often viewed as the gold standard. However, there is a variety of easy accessible sources for MSC-like cells such as adipose, synovial or perinatal tissue which offer a comparable differentiation capacity while causing minimal donor site morbidity [5, 19]. Therefore, the goal of this in vitro-study was to contribute further to this subject by proving the presence and comparing the characteristics of BM-MSCs and MSC-like cells isolated from arthritic articular cartilage, full-thickness samples of the joint capsule and the ligamentum capitis femoris (LCF) from the human arthritic hip joint.

## Methods

### Isolation and cultivation of cells

As described earlier, after informed written consent and as approved by University of Wuerzburg‘s institutional review board tissue samples for the isolation of cells were harvested from five female patients aged 40 to 55 (mean age 49 years), who underwent total hip arthroplasty [20, 21]. The donor tissues for the isolation of cells included femoral bone marrow reaming, arthritic femoral cartilage, full-thickness samples of the hip joint’s anterior capsule and the LCF and were extracted from each of the five patients´ hip joints during total hip replacement surgery. Total hip arthroplasty was performed using the anterior approach. All of the mentioned tissue samples are routinely removed with the implementation of this surgical method. Detailed descriptions of the location and extraction method for each donor tissue are listed in Table 1 (Table 1).

**Table 1:** Detailed description of the four different donor tissues used for the isolation of BM-MSCs and MSC-like cells

	Donor tissue	Surgical extraction
**BM-MSCs**	Bone marrow of the proximal femur	In order to fix the artificial shaft of the hip prothesis in the proximal femur, reaming of the proximal femoral shaft is necessary. The bone marrow cylinder removed during this procedure was secured.
**MSC-like cells**	Arthritic hyaline cartilage of the femoral head	The femoral head is removed during total hip arthroplasty. Macroscopically eroded, hyaline cartilage was scraped off the femoral head using a scalpel.
	Full-thickness samples of the anterior joint capsule	The hip joint was accessed through the anterior joint capsule. The full-thickness sample of the joint capsule removed during this procedure was secured.
	Ligamentum capitis femoris (LCF)	The LCF was attached to the femoral head which is removed during total hip arthroplasty. The LCF was removed from the femoral head using a scalpel.

Precise description of the four different donor tissues that were harvested during hip replacement surgery and were later used for the isolation of BM-MSCs and MSC-like cells from hyaline cartilage, the LCF and the hip joint’s anterior capsule

About 4 cm^3^ of each tissue was divided into small sized pieces of 1–2 mm^3^ in order to isolate cells. To optimize tissue breakdown arthritic hyaline cartilage was pre-digested using pronase E (0.2 mg/mL; Sigma-Aldrich, Schnelldorf, Germany) in Dulbecco’s Modified Eagle Medium (DMEM)/Ham’s F12 for 1 hour (1:1; Life Technologies GmbH, Thermo Fisher Scientific, Waltham, Massachusetts). Tissue samples derived from arthritic hyaline cartilage, the joint capsule and the LCF were then digested over night using collagenase (0.175 U/mL; Serva Electrophoresis, Heidelberg, Germany) in DMEM/Ham’s F12. In order to isolate cells from bone marrow, tissue samples were washed in in DMEM/Ham’s F12. The suspended cells were then spun, resuspended and seeded in 175 cm^2^ plastic cell culture flasks (Greiner Bio-One GmbH, Frickenhausen, Germany). The plastic adherent cells were grown in standard culture medium consisting of DMEM/Ham’s F12 supplemented with 10% fetal bovine serum (FBS) and 1% penicillin/streptomycin (PS) (all Life Technologies, Thermo Fischer Scientific, Dreieich, Germany). After reaching confluency, the cells were trypsinated and cryopreserved in liquid nitrogen. All samples were then stored in cryo tubes (Greiner Bio-One Gmbh) at − 80 °C for later experiments, resuspended in a medium consisting of 50% FBS, 40% DMEM/Ham’s F12 and 10% dimethylsulfoxid (DMSO) (AppliChem GmbH, Darmstadt, Germany). After harvesting the required cell samples from all five patients, the cells were thawed and examined in the following experiments under uniform conditions.

### FACS analysis of surface antigens

FACS analysis was performed on cells from bone marrow, arthritic cartilage, the joint capsule and the LCF of a single representatively selected patient. Cells were washed in phosphat buffered saline (PBS), spun and resuspended in standard cell culture medium. Cells were then counted and seperated into two 2,5 mL eppendorf tubes (Greiner Bio-One GmbH) per tissue sample. Each of the two eppendorf tubes contained a minimum amount of 5 × 10^5^ cells per tissue sample. After this, 100 μL of an antibody-PBS mixture containing a PBS/1% FBS pre-mix, CD73 PerCP antibody, CD44 eFLuor antibody, CD105 APC antibody and CD90 FITC antibody (all Thermo Fisher Scientific GmbH, Dreieich, Germany) was added to one of these two samples for fluorescence-activated cell sorting (FACS) analysis. The other sample was provided solely with 100 μL of PBS/1% FBS pre-mix and was therefore treated as a negative control. Both samples were then vortexed, stored in the dark (4 °C for 30 min), washed in PBS/1% FBS, spun and resuspended in 2% paraformaldehyde in PBS before being stored in the dark again (4 °C for 15 min). Finally, the cells were washed in PBS, spun, resuspended in PBS/1% FBS and vortexed. The two samples were then ready for FACS analysis which was performed with the BDTM LSR II X device (BD biosciences, Franklin Lakes, NJ, USA) and analyzed using the FlowJo 10.5.3 Software (FlowJo LLC, Ashland, OR, USA).

### Adipogenic and osteogenic differentiation

Multipotent differentiation capacity was evaluated in isolated cells derived from all four donor tissues harvested from the five patients´ hip joints. The cells stored at − 80 °C were thawed and resuspended in standard cell culture medium in plastic cell culture bottles until reaching confluency. The cells were then briefly trypsinated, counted and seeded in six-well plates (Greiner Bio-One GmbH, Frickenhausen, Germany) at a density of 3 × 10^3^ cells per cm^2^. Aditionally, cells were seeded in two-chamber slides for immunohistochemical stainings (Greiner Bio-One GmbH, Frickenhausen, Germany) at a density of 5 × 10^3^ cells per chamber. Cell cultures were stored at 37 °C, 5% CO_2_ and medium changes were performed every 3 to 4 days (d). After the cells showed confluent growth, osteogenesis and adipogenesis were induced as described in previous studies [22, 23]. Cells in other wells and chambers were grown in standard culture medium in order to serve as controls for comparison to the differentiated cells. The cell cultures were fixed after 21 d.

Stainings were performed as reported previously in order to detect mineralization of the extracellular matric (ECM) using Alizarin Red S (1%; Sigma-Aldrich) [23], while immunohistochemical stainings were used to reveal extracellular collagen type I (Col I) formations. The adipogenic differentiation was detected through staining of lipid drops using Oil RedO (all Sigma-Aldrich) as described previousely [24]. In addition, the gene expression levels of the tissue specific osteogenic marker genes alkaline phosphatase (ALP), Col I, collagen type X (Col X) or osteocalcin (OC), as well as the adipogenic marker genes lipoproteinlipase (LPL) and peroxisome proliferator-activated receptor γ (PPARγ) were examined using RT-PCR.

### Chondrogenic differentiation

In order to confirm the multilineage differentiation potential of isolated cells from all five patients further, their chondrogenic differentiation potential was tested using pellet culture in centrifuge tubes as described previously [25]. Pellets were grown in chondrogenic differentiation medium as reported earlier [22]. Four pellets per tissue sample were formed for chondrogenic differentiation while four pellets were maintained in a control medium missing transforming growth factor (TGF)-ß1. The cells were then centrifuged to promote aggregate formation and stored at 37 °C, 5% CO_2_, while medium changes were performed every 3 to 4 d. After 27 d of culture the pellets were placed in Tissue-Tek® Cryomold® Standard (Sakura Fintek, Torrance, CA, USA) before being cryopreserved in liquid nitrogen and stored at − 80 °C. The frozen pellets were sectioned at 6 μm thickness and placed on SuperFrost® cryosection slides (Thermo Fischer Scientific GmbH). To fix the cells the pellets were covered in 3% acetic acid (3 min; Carl Roth GmbH). Alcian blue (1%, Sigma-Aldrich) stainings were performed to verify the formation of proteoglycans as described earlier [26]. Immunohistochemical stainings were used to detect collagen type II (Col II) and Col X. The expression of chondrogenic markers aggrecan (AGG), Col II and sex-determining region Y-box 9 (Sox-9) was examined using RT-PCR.

### Histology and immunohistochemistry

Monolayers were fixed in ice-cold methanol, while pellet cultures were placed in Tissue-Tek® Cryomold® Standard (Sakura Fintek, Torrance, CA, USA) before being cryopreserved in liquid nitrogen. The frozen pellets were sectioned, placed on SuperFrost® cryosection slides (Thermo Fischer Scientific GmbH) and fixed with 3% ice-cold acetone (Carl Roth GmbH). Alizarin Red S, Oil redO and alcian blue stainings were performed as outlined previously [23, 24, 26]. In addition, immunohistochemical stainings were performed on monolayers and pellets using the following antibodies: Col I - monoclonal anti Col Iα1 (5 μg/mL; Abcam pls, Cambridge, Great Britain); Col II - polyclonal Col IIα1 antibodies (5 μg/mL; Acris Antibodies GmbH, Herford, Germany); Col X - polyclonal Col X antibodies (5 μg/mL; Abcam pls). The immunostainings were visualised with the Avidin-Biotion complex method using the protocols, biotinylated antibodies, blocking serum and peroxidase from the VECTASTAIN® Universal Elite® ABC Kit (Vector Laboratories, Burlingame, CA, USA) and the VECTOR® NovaRED™ peroxidase substrate kit (Vector Laboratories). The slides and wells were counterstained with haematoxylin (Sigma-Aldrich). For control stainings the primary antibodies were replaced with non-immune IgG antibodies (Sigma-Aldrich).

### RNA isolation and semiquantitative RT-PCR analysis

RNA was isolated from control and from differentiated samples of adipogenic, osteogenic (21 d) and chondrogenic (27 d) differentiated cell cultures using Trizol reagent (Invitrogen) and further purification steps including DNase treatment as described in the user’s guide (NucleoSpin® RNA II kit, Macherey-Nagel GmbH & Co. KG, Düren, Germany). To create cDNA 1 μg of purified RNA was combined with random hexamer primers (Thermo Fischer Scientific) and Promega® M-MLV reverse transcriptase (Promega GmbH, Mannheim, Germany). 1 μL of cDNA served as a pattern for amplification in a 30 μL reaction volume containing GoTaq® DNA polymerase (Promega GmbH) with forward and reverse gene-specific primers (5 pmol each). The primers and their specific sequences, annealing temperatures and cycle numbers are listed in Table 2. As described earlier Elongation factor 1α (EEF 1α) was used as the housekeeping gene [22, 27]. The final products of RT-PCR were seperated through electrophoresis on 2% agarose (Biozym Scientific GmbH, Hessisch Oldendorf, Germany) gels containing 5 μL per 100 mL GelRed® (Biotium, Fremont, CA, USA). The band densities for each primer pair were then examined to measure the mean ratio and standard deviation for all of the tested genes in comparison to the expression of the EEF 1α housekeeping gene.

**Table 2:** Primer details for semiquantitative RT-PCR

Gene	Primer sequences (5′-3′)	Annealing temperature (°c)	Product size (base pairs)	Cycles	MgCl_**2**_
*Housekeeping gene for internal control*					
EEF1α	Sense: AGGTGATTATCCTGAACCATCC Antisense: AAAGGTGGATAGTCTGAGAAGC	54.0	234	21	1 x
*Adipogenic marker genes*					
LPL	Sense: GAGATTTCTCTGTATGGCACC Antisense: CTGCAAATGAGACACTTTCTC	51.0	239	30	1x
PPARγ	Sense: GCTGTTATGGGTGAAACTCTG Antisense: ATAAGGTGGAGATGCAGGCTC	61.0	297	35	1x
*Osteogenic marker genes*					
Col I	Sense: GGACACAATGGATTGCAAGG Antisense: TAACCACTGCTCCACTCTGG	55.0	461	22	2x
Col X	Sense: CCCTTTTTGCTGCTAGTATCC Antisense: CTGTTGTCCAGGTTTTCCTGGCAC	54.0	468	40	1x
ALP	Sense: TGGAGCTTCAGAAGCTCAACACCA Antisense: ATCTCGTTGTCTGAGTACCAGTCC	51.0	454	33	1x
OC	Sense: ATGAGAGCCCTCACACTCCTC Antisense: GCCGTAGAAGCGCCGATAGGC	62.0	293	35	2x
*Chondrogenic marker genes*					
AGG	Sense: GCCTTGAGCAGTTCACCTTCAntisense: CTCTTCTACGGGGACAGCAG	54.0	400	35	1x
Col II	Sense: TTTCCCAGGTCAAGATGGTC Antisense: CTTCAGCACCTGTCCACCA	51.0	155	31	1x
Sox-9	Sense: ATCTGAAGAAGGAGAGCGAG Antisense: TCAGAAGTCTCCAGAGCTTG	60.0	263	31	1x

*EEF 1α* Elongation factor 1α, *LPL* Lipoproteinlipase, *PPARγ* Peroxisome proliferator-activated receptor γ, *Col I* Collagen type I, *Col X* Collagen type X, *ALP* Alkaline phosphatase, *OC* Osteocalcine, *AGG* Aggrecane, *Col II* Collagen type II, *Sox-9* Sex-determining region Y-box 9

### Statistical analysis

Semiquantitative RT-PCR experiments were performed on four different tissues each taken from five different donors (*n* = 5) and expressed as mean values ± standard deviation. Statistical significance was defined using the Mann-Whitney-U-Test with *p* < 0,05 being considered as significant.

## Results

### Surface markers on isolated cells

**Fig. 1:**
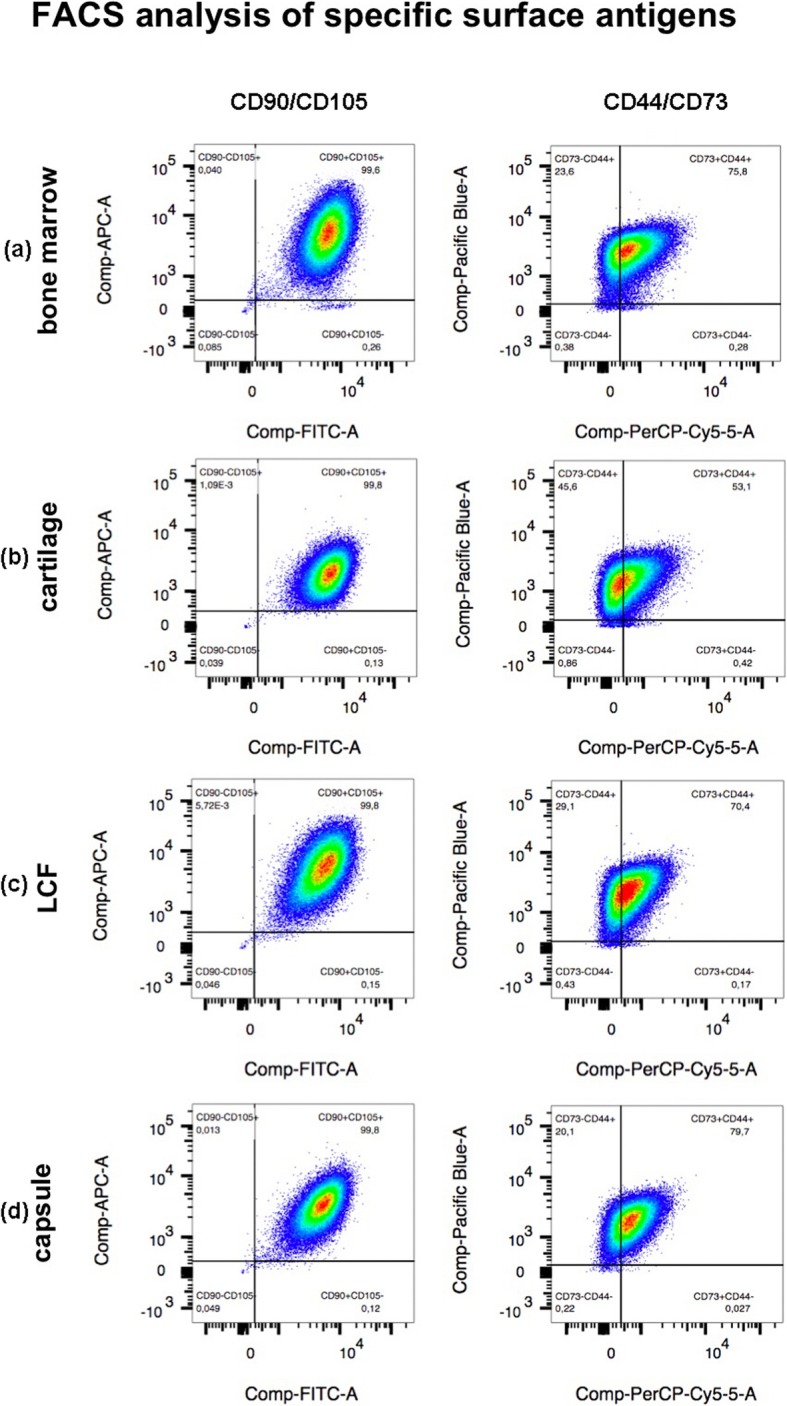
Fluorescence-acitvated cell sorting (FACS) analysis of the expression of surface antigens on cells isolated from all four donor tissues of one single patient. A minimum amount of 5 × 10^5^ cells from bone marrow (**a**), hyaline cartilage (**b**), the LCF (**c**) and the joint capsule (**d**) were examined for the (co-)expression of surface antigens CD44, CD73 (CD44/CD73), CD90 and CD105 (CD90/CD105). The results were pictured using the FLowJo 10.5.3 Software by FlowJo LLC. While almost all cells (≥ 95%) were positive for the surface markers CD44, CD90 and CD105 the percentage of CD73^+^ cells ranged from about 53–80% depending on the donor tissue. CD, cluster of differentiation. LCF, ligamentum capitits femoris

The expression of the surface antigens CD44, CD73, CD90 and CD105 on cells derived from the four different donor tissues of a single, representatively chosen patient were examined using FACS analysis (Fig. 1). While the co-expression of CD73, CD90 and CD105 is regarded as highly characteristic for MSCs, this non-standard panel does not examine the presence of haematopoetic markers as requested in the ISCT’s criteria for the definition of MSCs. All isolated cells from bone marrow, cartilage, LCF and the joint capsule were strongly positive (≥ 95%) for CD44, CD90 and CD105. The majority of isolated cells was also strongly positive (< 95%) for the surface marker CD73. While > 70% of cells isolated from bone marrow, cartilage and the joint capsule expressed CD73, only about half of the cells from the LCF were positve for this surface marker. Matching results were shown within the coexpression of CD-antigens (Fig. 1).

### Histological analysis of adipogenic differentiation

**Fig. 2:**
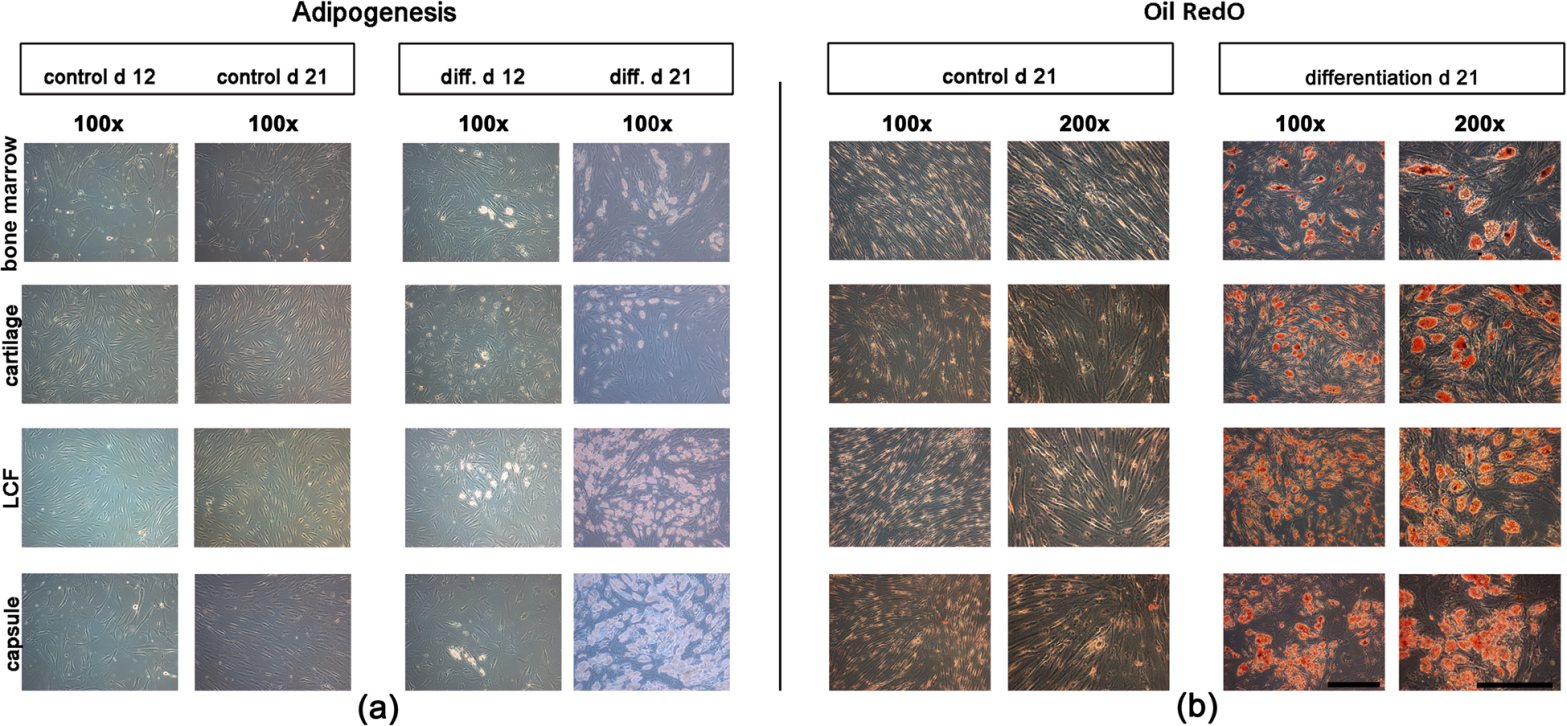
Assay of adipogenesis in mesenchymal progenitor cells after 21 days in monolayer cell culture. For inducing adipogenesis monolayer cultures containing mesenchymal progenitor cells from bone marrow, hyaline cartilage, LCF and the joint capsule were incubated with adipogenic differentiation medium for 21 days. Controls were maintained in cell culture medium. Both native, unstained tissue samples (**a**) and Oil RedO stainings (**b**) from control and differentiatied samples were compared. In contrast to controls the formation of lipid droplets increased during day 12 to day 21 of adipogenesis (**a**) and all differentiated cultures stained positive for Oil Red O at the end of the differentiation period (**b**). Representative samples were captured at low (100x; black bar = 200 μm) and high (200x; black bar = 150 μm) magnification. d, days; diff., differentiation; LCF, ligamentum capitits femoris

In comparison to control cultures (Fig. 2, b, control d 21) all cells derived from bone marrow, cartilage, the joint capsule and the LCF which were incubated in an adipogenic differentiation medium for 21 d showed positive Oil RedO stainings (Fig. 2, b, differentiation d 21). The quantity of lipid droplets in all adipogenic differentiated cultures increased from d 12 (Fig. 2, a, diff. d 12) to d 21 (Fig. 2, a, diff. d 21).

### Adipogenic marker gene expression after 21 d

**Fig. 3:**
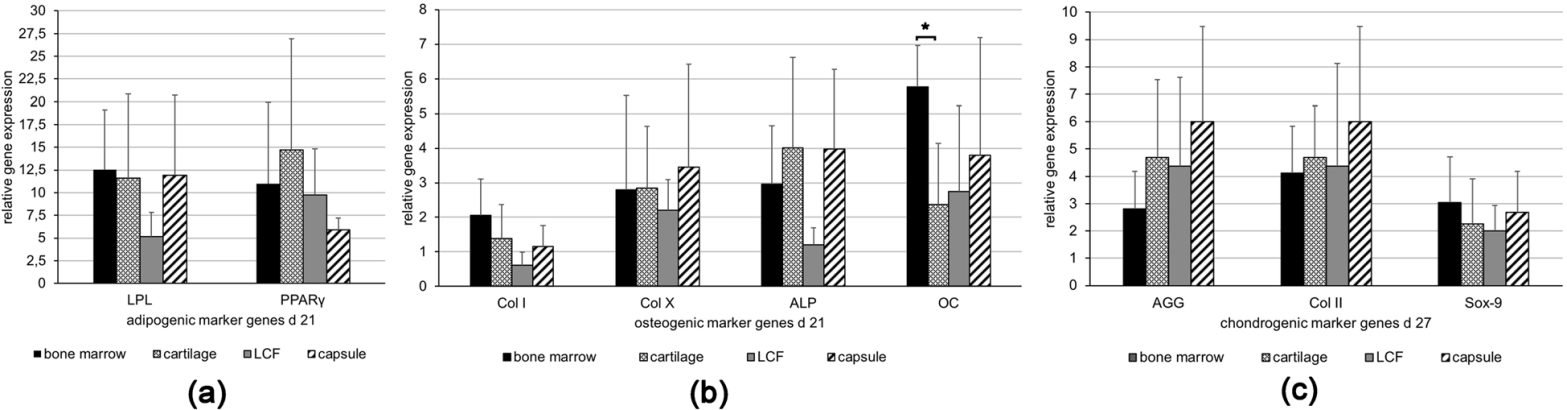
Mean changes in the expression of adipogenic, osteogenic and chondrogenic marker genes ± standard deviation as measured by semiquantitative RT-PCR in mesenchymal progenitor cells at the end of the respective differentiation period. Mesenchymal progenitor cells derived from bone marrow, hyaline cartilage, LCF and the joint capsule were incubated with adipogenic (21 d), osteogenic (21 d) and chondrogenic (27 d) differentiation medium. The mean changes in the relative expression of the adipogenic (**a**) marker genes - lipoproteinlipase (LPL) and peroxisome proliferator-activated receptor γ (PPARγ) - the osteogenic (**b**) marker genes - collagen type I (Col I), collagen type X (Col X), alkaline phosphatase (ALP) and osteocalcin (OC) - as well as the chondrogenic (**c**) marker genes - (AGG), collagen type II (Col II) and sex-determining region Y-box 9 (Sox-9) - are pictured, respectively. Error bars picture the range of changes in the mean expression of specific marker genes in differentiated cell cultures from which the standard deviations were calculated. Elongation factor 1α (EEF 1α) was used as the housekeeping gene and for internal controls. Primer details are illustrated in Table 1. d, days; LCF, ligamentum capitits femoris

After 21 d the adipogenic differentiated cultures from all four donor tissues showed a similar, clear up-regulated mean value of mRNA expression levels of adipogenic marker genes lipoproteinlipase (LPL) and peroxisome proliferator-activated receptor γ (PPARγ) in comparison to their controls (Fig. 3, a). The mean expression levels of LPL in cells from LCF (Fig. 3, a, LPL) and PPARγ in MSC-like cells derived from the joint capsule (Fig. 3, a, PPARγ) were lower than in cartilage or bone marrow-derived cells, although not significantly. The pictured error bars show a high standard deviation regarding the relative changes in gene expression of LDL and PPARγ in differentiated cultures and therefore indicate a strong diversification of results for the five different patients (Fig. 3, a).

### Histological and immunohistochemical analysis of osteogenesis

**Fig. 4:**
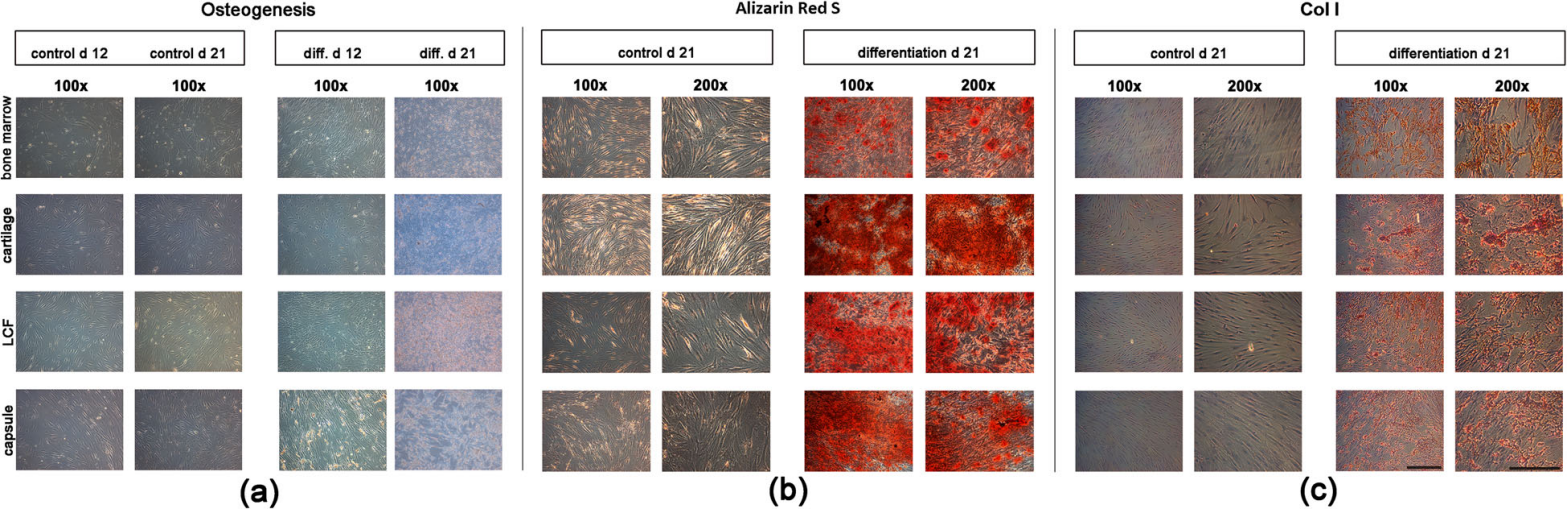
Histological and immunohistochemicasl assay of osteogenesis in cells after 21 days in monolayer cell culture. For inducing osteogenesis monolayer cultures containing BM-MSCs and MSC-like cells from hyaline cartilage, LCF and the joint capsule were incubated with osteogenic differentiation medium for 21 days. Contols were maintained in cell culture medium. Native, unstained tissue samples (**a**), Alizarin Red S stainings (**b**) for detection of extracellular calcium deposits and immunohistochemical stainings of collagen type I (Col I; **c**) from control and differentiatied samples were compared. From day 12 to day 21 dense deposits formed in differentiated monolayers and Alizarin Red S (**a**) as well as immunohistochemical stainings of Col I (**c**) were positive in all differentiated cell cultures in contrast to controls. Representative samples were captured at low (100x; black bar = 200 μm) and high (200x; black bar = 150 μm) magnification. Col I, collagen type I; d, days; diff., differentiation; LCF, ligamentum capitits femoris

In all monolayer cultures incubated in osteogenic differentiation medium spider web-like cell patterns, surrounded by an increasing amount of extracellular, dense deposits formed from d 12 (Fig. 4, a, diff. d 12) to d 21 (Fig. 4, a, diff. d 21). After 21 d positive Alizarin Red S staining identified these dense depostis as calcium aggregates in all osteogenic differentiated cell-cultures from bone marrow, cartilage, the joint capsule and LCF (Fig. 4, b, differentiation d 21). In addition, all osteogenic differentiated cultures showed a clearly positive immunohistochemical staining for Col I at the end of the differentiation period after 21 d (Fig. 4, c, differentiation d 21). Controls remained negative for both Alizarin Red S (Fig. 4, b, control d 21) and immunohistochemical stainings (Fig. 4, c, control d 21).

### Osteogenic marker gene expression after 21 d

RT-PCR revealed an upregulation of the mean mRNA expression levels of osteogenic marker genes collagen type X (Col X), alkaline phosphatase (ALP) and osteocalcin (OC) in all the osteogenic differentiated cultures compared to their controls (Fig. 3, b). The expression of the osteogenic marker gene OC in BM-MSCs was significantly higher (*p* < 0,05) than in MSC-like cells isolated from cartilage (Fig. 3, b, OC). Interestingly, the mean expression of Col I mRNA only showed a clear upregulation in BM-MSCs (Fig. 3, b, Col I). Otherwise, the expression of osteogenic marker genes did not differ significantly within MSC-like cells derived from different native tissues. The pictured error bars show a high standard deviation regarding the relative changes in gene expression for all osteogenic marker genes and therefore indicate a strong diversification of results for the five different patients (Fig. 3, b).

### Histological and immunohistochemical analysis of chondrogenesis

**Fig. 5:**
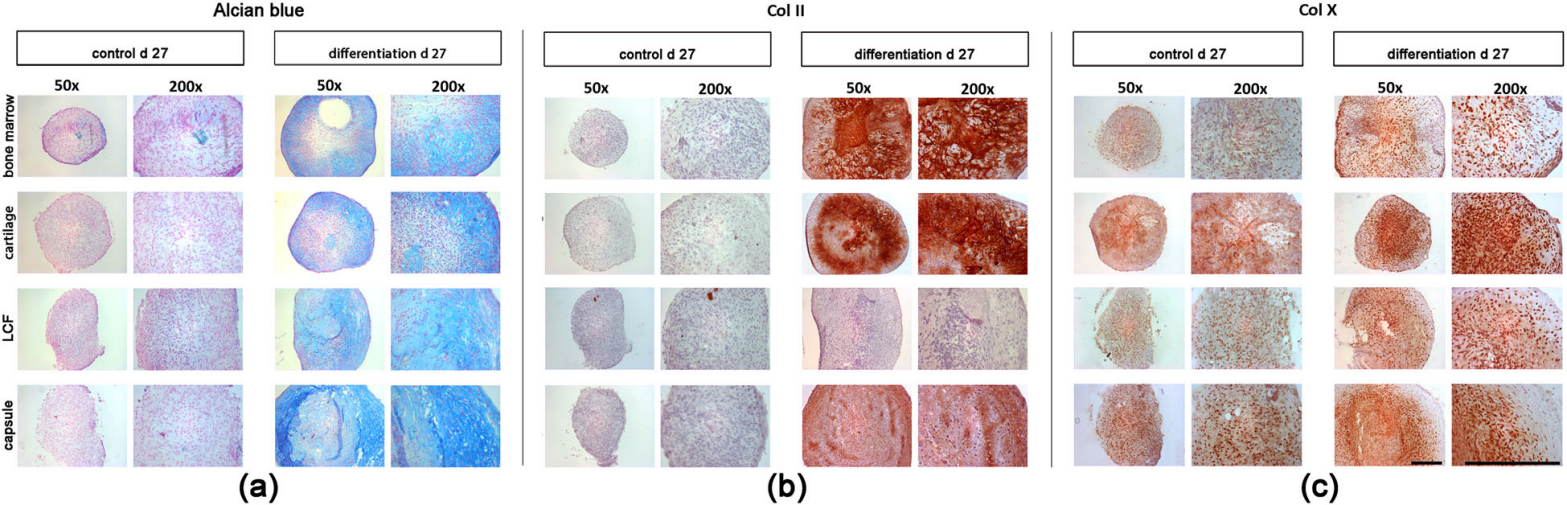
Histological and immunohistochemical analysis of chondrogenesis in cell-pellets after 27 d of pellet culture in presence of transforming growth factor-ß1. For inducing chondrogenesis pellets containing BM-MSCs and MSC-like cells from hyaline cartilage, LCF and the joint capsule were incubated with chondrogenic differentiation medium for 27 days. Contols were maintained in cell culture medium. After 27 days alcian blue staining (**a**) was performed for the detection of proteoglycans. While the controls remained negative all differentiated pellets showed positive alcian blue stainings. Immunohistochemical stainings of collagen type II (Col II; **b**) and collagen type X (Col X; right panel) were performed on pellet sections containing mesenchymal progenitor cells from bone marrow, hyaline cartilage (**b**), the LCF (**c**) and the joint capsule (**d**) after incubation in chondrogenic differentiation medium for 27 days. Controls were maintained in cell culture medium for the same duration of time (left panel). Positive staining for collagen type II (Col II; b) appeared red. Col II was detected in all differentiated pellet sections except from LCF (**b**, LCF), while moderate collagen type X (Col X; c) stainings were shown in all differntiated pellet sections and their controls after 27 days (**c**). Representative samples were captured at low (50x; black bar = 300 μm) and high (200x; black bar = 300 μm) magnification. Col II, collagen type I; Col X, collagen type X; d, days; diff., differentiation; LCF, ligamentum capitits femoris

After 27 d of pellet-culture in the presence of TGF-ß1, positive alcian blue stainings indicated the presence of proteoglycans in all chondrogenic differentiated cell-pellets derived from bone marrow, cartilage, the joint capsule and LCF (Fig. 5, a, differentiation d 27). In addition, chondrogenic differentiated pellets containing cells from bone marrow and cartilage showed a chondrogenic phenotype particularly in outer regions of the pellets (Fig. 5, a, bone marrow, cartilage). Despite clearly postive alcian blue stainings, hyaline cartilage-specific attributes, such as chondrones in areas with a low density of cells, were less visible in pellets from the joint capsule (Fig. 5, a, capsule) and almost non-existent in pellets from LCF (Fig. 5, a, LCF). Corresponding to alcian blue stainings, immunohistochemical stainings revealed vast amounts of collagen type II (Col II) in outer regions of the differentiated pellets from bone marrow, cartilage and the joint capsule after 27 d (Fig. 5, b, differentiation d 27). In comparison to the strong immunohistochemical stainings of Col II in differentiated pellets from bone marrow and cartilage (Fig. 5, b, bone marrow, cartilage), the staining appeared less intense in differentiated pellets from the joint capsule (Fig. 5, b, capsule). In contrast, chondrogenic differentiated pellets from LCF showed no visible stainings of Col II after 27 d (Fig. 5, b, LCF). All controls remained negative for alcian blue (Fig. 5, a, control d 27) and Col II (Fig. 5, b, control d 27) stainings after 27 d of pellet culture in absence of TGF-ß1.

Immunohistochemical stainings for Col X were performed to examine possible chondrocyte hypertrophy during chondrogenic differentiation (Fig. 5, c). While controls remained mostly negative (Fig. 5, c, control d 27), differentiated pellets showed slightly stronger stainings especially in cell-rich inner regions of the sections (Fig. 5, c, differentiation d 27) that showed a less chondrogenic phenotype in earlier alcian blue and Col II stainings. The strongest matrix staining for Col X after 27 d was seen in the differentiated pellets containing cells from cartilage (Fig. 5, c, cartilage).

### Expression of chondrogenic marker genes after 27 d of pellet-culture

RT-PCR showed an upregulation of the mean mRNA levels of the examined chondrogenic marker genes AGG, Col II and Sox-9 in all chondrogenic differentiated pellets after 27 d (Fig. 3, c). The mean mRNA expression levels of AGG and Col II were highest in chondrogenic differentiated pellets from the joint capsule, although observed differences were not from statistical significance (Fig. 3, c, AGG, Col II). Interestingly, the mean expression of Col II mRNA was upregulated in differentiated pellets from LCF (Fig. 3, c, Col II), although the immunohistochemical stainings remained negative for Col II in corresponding pellets from LCF (Fig. 3, b, LCF). Significant differences in the mean mRNA expression levels between the pellets from varying native tissues were not observed. The pictured error bars show a high standard deviation regarding the relative changes in gene expression for all chondrogenic marker genes in differentiated pellets and therefore indicate a strong diversification of results for the five different patients (Fig. 3, c).

## Discussion

MSC-like cells isolated from easy accessible tissues, such as adipose or synovial tissue, have shown to possess a potentially superior differentiation capacity compared to BM-MSCs and thus sparked great interest among clinicians and researchers [19, 28]. The goal of our study was to contribute further to this subject by detecting and examinating potential MSC-like cells in the LCF and full-thickness samples of the joint capsule of human arthritic hip joints in five female patients undergoing total hip arthroplasty. The in vitro characteristics of isolated cells were compared to the well researched populations of BM-MSCs and MSC-like cells isolated from arthritic hyaline cartilage simultaneously derived from the same patient’s hip joints.

First and formost, our study showed that cells could be isolated from bone marrow, arthritic hyaline cartilage, the LCF and full-thickness samples of the joint capusles of the five patients´ examined arthritic hip joints which grew plastic-adherent and showed multipotent differentiation capacity. In addition, the cells from all four donor tissues harvested from one representatively selected patient clearly co-expressed the surface antigens CD44, CD73, CD90 and CD105 independant of the investigated donor tissue. In accordance with our findings, the presence of BM-MSCs, which most likely reside within the endosteal or endovascular lining of cells [24, 29, 30], and MSC-like cells found in the superficial zone of arthritic or healthy hyaline cartilage [31, 32] has frequently been confirmed by earlier in vitro-studies. While MSC-like cells have also previously been obtained from the joint capsule of the human hip, interestingly, the tissue utilized for the isolation of cells was exclusively derived from the synovial stratum [33]. Within the synovial stratum MSC-like cells are believed to reside within the intimal layer of the synovial membrane [34]. In addition, there is no data exclusively describing the characterization of MSC-like cells isolated from the LCF. However, our group and Cheng et al. described the isolation of MSC-like cells from the anterior cruciate ligament (ACL) of the knee in earlier studies [22, 35]. Cheng et al. showed that CD44, CD90 and CD105 positive, MSC-like cells were located in fascicles and the endothelial lining within sections of the ACL [35]. Furthermore, more recent studies showed that MSC-like cells which are positive for CD73 and CD90 may also be located in sinusoids close to the surface of the ACL [36]. Eventhough the two ligaments are not identical, the ACL and the LCF share various histological and anatomical similarities and both form intraarticular ligaments in synovial joints coated by a synovial membrane [37]. Both of these findings support the complementary and novel results found in our study in which MSC-like cells could be isolated from the LCF and full-thickness tissue samples including the ligamentous tissue of the fibrous stratum of the arthritic hip’s joint capsule. Moreover, all four tissues examined in our study originate from the LPM and are well vascularized, making them home to pericytes. This supports our results when taking more recent studies into consideration which discuss the embryonic developement of MSCs [38]. Healthy hyaline cartilage represents an exception since it is avascular. This led to previous debates on whether multipotent precursors, which have been shown to be located close to the surface in arthritic and healthy hyaline cartilage, may be distinct from MSCs or if precursors other than PSCs contribute to the formation of MSCs [38, 39]. On the other hand, blood vessels from the subchondral in osteoarthritic joints bone can infiltrate the calcified layers of arthritic hyaline cartilage and potentially deliver MSC-like cells [40].

Secondly, we examined cells derived from one exemplary selected patient sample regarding the expression of the surface markers CD44, CD73, CD90 and CD105 to highlight potential differences within the four selected donor tissues. We found that ≥95% of the examined cells derived from bone marrow, arthritic hyaline cartilage, the joint capsule and the LCF showed a clearly positive co-expression of surface antigens CD44, CD73, CD90 and CD105 as shown through FACS analysis. Although a large number of isolated cells were also positive for CD73, this fraction did not exceed 95% independent of the type of donor tissue. The expression of CD73 was not remarkably distinct within any of the examined donor tissues, which may indicate that staining with different antibody conjugates could have resulted in ≥95% for all surface markers. Although this non-standard panel does not meet the ISCT’s requirements for MSCs since the presence of haematopoetic surface antigens was not examined, it shows that all tested cells isolated from the four donor tissues of the exemplary selected patient carried a compilation of surface antigens which is viewed as highly characteristic but not specific for MSCs [18].

The presence of these surface markers on BM-MSCs has previously been proved in various studies [29, 41–43]. Alsalameh et al. and Pretzel et al. also showed that MSC-like cells derived from healthy and arthtitic cartilage, co-express the surface markers CD105 and CD166 [31, 32]. Multiple researchers, including ourselves, also isolated MSC-like cells expressing the surface antigens CD73, CD90 and CD105 from the ACL [22, 35] or the synovial membrane and synovial fluid of the hip joint’s capsule [19, 44, 45]. This strongly supports our findings in which cells expressing the surface markers CD44, CD73, CD90 and CD105 can also be found in full-thickness samples of the joint capsule and LCF of the human hip joint.

Thirdly, mulitpotent differentiation potential varied but was proven for BM-MSCs and MSC-like cells isolated from arthritic cartilage, the joint capsule and LCF of the human hip joint as requested by the ISCT’s minimal criteria for MSCs [17]. Adipogenic and osteogenic differentiation capacity, as proven by histological, immunohistochemical and molecularbiological analysis, showed hardly any visual differences between the cells of different donor tissues. It must be taken into account that no quantitative measurements were made. Interestingly, the clearly positive immunohistochemical stainings of Col I in all osteogenic differentiated samples (Fig. 4, c) did not match the irregular upregulation of the expression of Col I at the mRNA level in the matching osteogenic differentiated cell cultures (Fig. 3, b). This could reflect the phaseal upregulation of Col I during osteogenic differentiation which can lead to punctual differences in protein synthesis and gene regulation. Lastly, chondrogenic differentiation was induced using a TGF-ß1 based differentiation medium and pellet culture system as described earlier by Johnstone et al. [46]. Three dimensional culture systems provide mechanical stimuli, similar to the enchondral ossification in the human growth plate, that are necessary for chondrogenic differentiation in vitro [47–49]. Although pellets from all four donor tissues showed successfull chondrogenesis in histological and molecularbiological analysis, immunohistochemical stainings of Col II (Fig. 5, b) indicated a superior chondrogenic differentiation capacity of BM-MSCs and MSC-like cells isolated from arthritic hyaline cartilage.

The trilineage differentiation capacity of BM-MSCs using similar methods as used in this study has been repeatedly validated [24, 29]. Because bone marrow was the first discovered source for the isolation of MSCs in vitro, it is often reffered to as the gold standard to which other MSC-like cells are compared to. In line with the results of our study Alsalameh et al. and Pretzel et al. showed that the adipogenic, osteogenic and chondrogenic differentiation capacity of MSC-like cells derived from arthritic hyaline cartilage of the knee did not differ significantly from that of BM-MSCs [31].

Segawa et al. found that MSC-like cells derived from synovial and subsynvoial tissue of the medial capsule of the knee joint showed similar adipogenic and osteogenic differentiation capacity compared to BM-MSCs [19]. Interestingly, the chondrogenic differentiation potential of these synovial derived MSC-like cells was decleared superior to that of BM-MSCs as measured in mean size and dry weight of chondrogenic differentiated pellets [19]. Another study by Hatakeyama et al. compared the multipotent differentiation capacity between synovial derived MSC-like cells from the hip and the knee joint. The results showed that while adipogenic and osteogenic differentiation capacity was significantly higher for cells isolated from the knee joint, their chondrogenic differentiation potential was similar [33]. Although we used both, the synovial stratum and the ligamentous tissue from the fibrous stratum for the isolation of cells from the hip’s joint capsule, these findings correspond to our results in which the multipotent differentiation capacity of joint capsule-derived MSC-like cells was mostly comparable to that of BM-MSCs. Unfortunately, pellet size and dry weight of differentiated pellets were not measured in our study, making a direct comparison to earlier research not possible. Nevertheless, the chondrogenic phenotype as well as the intensity of immunohistochemical stainings of Col II in differentiated pellet-sections containing BM-MSCs were superior to those containing cells from the joint capsule.

As mentioned above, there is no scientific data about the isolation and multipotent differentiation capacity of MSC-like-cells from the LCF. Similar to the LCF in the hip, the ACL forms an intraarticular, vascularized ligament in the knee. Although, this does not allow MSC-like cells derived from both tissues to be regarded as equivalent we compared our findings for MSC-like cells derived from the LCF to those from studies that isolated MSC-like cells from the ACL [22, 35]. Matching the results of the trilineage differentiation of MSC-like cells derived from the LCF in our recent study, Steinert et al. found that the BM-MSCs possessed greater chondrogenic differentiation capacity compared to MSC-like cells isolated from the ACL, while their osteogenic and adipogenic differentiation potential did not differ significantly [22]. In contrast to our findings, Steinert et al. found positive immunohistochemical stainings of Col II in chondrogenic differentiated pellet-sections containing cells derived from the ACL in the presence of TGF-ß1 [22]. Although Cheng at al. found similar results regarding the adipogenic differentiation capacity of both sources of cells, they reported a higher osteogenic and similar chondrogenic differentiation capacity of BM-MSCs compared to the MSC-like cells derived from the ACL [35].

In summary, all cells derived from bone marrow, hyaline cartilage, the LCF and full-thickness samples of the joint capsule of five patients’ arthritic hip joints grew plastic-adherent and were capable of multipotent differentiation. FACS analysis, which was performed using cells derived from the four donor tissues of one exemplary chosen patient, showed that cells were clearly and equally positive for the surface antigens CD44, CD73, CD90 and CD105 which are viewed as highly characteristic for MSCs. This indicates that MSC-like cells, which are very similar to those found in bone marrow and hyaline cartilage of the hip joint, can also be isolated from the LCF and full-thickness samples of the hip joint’s capsule which are routinely removed during total hip replacement surgery. Important limitations to this hypothesis and our in vitro study include missing quantitative measurements of multipotent differentiation as well as the examination of surface antigens solely on cells derived from one of the five patients. Further, the non-standard panel of surface antigens did not include haematopoetic surface markers as requested by the ISCT. In addition, multiple studies showed that MSC-like cells isolated from different donor tissues require a varying composition of growth factors and scaffolds to optimize their multipotent differentiation potential [38, 50]. This has to be considered when comparing the chondrogenic differentiation potential of cells examined in our study and indicates that further research is necessary to fully determine suitable growth factors and fitting biomaterials to optimize the chondrogenic differentiation of MSC-like cells derived from full-thickness samples of the joint capsule and the LCF.

## Conclusions

All examined tissues of the arthritic hip joint - bone marrow, arthritic hyaline cartilage, LCF and the joint capsule - were proved to be home to multipotent, plastic-adherent growing and CD44, CD73, CD90, CD105 positive MSC-like cells. While the osteogenic and adipogenic differentiation potential showed no significant differences depending on the source of MSCs used, further research is necessary to fully determine the chondrogenic differentiation potential of MSC-like cells isolated from LCF and full-thickness samples of the hip’s joint capsule in vitro.

## Data Availability

The datasets used and analysed during the current study are available from the corresponding author on reasonable request.

## Abbreviations

ACL: Anterior cruciate ligament
AGG: Aggrecan
ALP: Alkaline phosphatase
BM-MSCs: Bone marrow-derived mesenchymal stromal cells
CD: Cluster of differentiation
cDNA: Complementary DNA
Col I: Collagen type I
Col II: Collagen type II
Col X: Collagen type X
d: Days
DMSO: Dimethylsulfoxid
ECM: Extracellular matrix
EEF1α: Elongation factor 1α
FACS: Fluorescence-activated cell sorting
FBS: Fetal bovine serum
H&E: Hematoxylin-Eosin
ISCT: International Society for Cellular Therapy
LCF: Ligamentum capitis femoris
LPL: Lipoproteinlipase
LPM: Lateral plate mesoderm
MPCs: Mesenchymal progenitor cells
MSCs: Mesenchymal stromal cells
OC: Osteocalcin
PBS: Phosphat buffered saline
PPARγ: Peroxisome proliferator-activated receptor γ
PS: Penicillin/streptomycin
PSCs: Perivascular stem cells
RT-PCR: Reverse transcriptase polymerase chain reaction
Sox-9: Sex-determining region Y-box 9
TGF: Transforming growth factor
